# Self-Powered, Flexible, Transparent Tactile Sensor Integrating Sliding and Proximity Sensing

**DOI:** 10.3390/ma18020322

**Published:** 2025-01-13

**Authors:** Kesheng Wang, Shouxin Du, Jiali Kong, Minghui Zheng, Shengtao Li, Enqiang Liang, Xiaoying Zhu

**Affiliations:** 1 School of Mechanical Engineering, Shandong Huayu University of Technology, Dezhou 253034, China; 2 Department of Equipment Maintenance and Remanufacturing Engineering, Academy of Army Armored Forces, Beijing 100072, China

**Keywords:** tactile sensor, sliding and proximity sensing, flexible and transparent device

## Abstract

Tactile sensing is currently a research hotspot in the fields of intelligent perception and robotics. The method of converting external stimuli into electrical signals for sensing is a very effective strategy. Herein, we proposed a self-powered, flexible, transparent tactile sensor integrating sliding and proximity sensing (SFTTS). The principle of electrostatic induction and contact electrification is used to achieve tactile response when external objects approach and slide. Experiments show that the material type, speed, and pressure of the perceived object can cause the changes of the electrical signal. In addition, fluorinated ethylene propylene (FEP) is used as the contact electrification layer, and indium tin oxide (ITO) is used as the electrostatic induction electrode to achieve transparency and flexibility of the entire device. By utilizing the transparency characteristics of this sensor to integrate with optical cameras, it is possible to achieve integrated perception of tactile and visual senses. This has great advantages for applications in the field of intelligent perception and is expected to be integrated with different types of optical sensors in the future to achieve multimodal intelligent perception and sensing technology, which will contribute to the intelligence and integration of robot sensing.

## 1. Introduction

Tactile perception is crucial in intelligent perception and robot perception, significantly enhancing the capabilities and functions of robots. Tactile sense, visual, and auditory sensing play a very important role in smart robots. Just like the human features, anyone is indispensable, and they are all important parameters in intelligent evaluation. Tactile sensors enhance the adaptability and versatility of robots, enabling them to adjust their actions based on the physical characteristics of objects, making them suitable for tasks in unstructured environments such as search and rescue. In the field of prosthetics, integrated tactile sensors can provide users with tactile sensation, significantly improving their quality of life. In addition, the integration of tactile sensors with other sensory modes, such as vision, forms a more powerful and comprehensive perception system, enabling robots to better understand and interact with their environment, especially in tasks that require multiple sensory inputs. For example, robots equipped with visual and tactile sensors can more accurately recognize and manipulate objects, even in poor visual conditions. In short, tactile perception is indispensable for the development of intelligent perception and robot perception. It improves the accuracy, adaptability, and versatility of robots, enabling them to perform complex tasks more efficiently and safely. With technological advancements, the integration of tactile sensors will play a crucial role in developing more autonomous and powerful robot systems.

Currently, proximity sensing in tactile perception generally includes capacitive [1,2,3], electromagnetic [4,5,6] methods. However, capacitive sensing is significantly affected by parasitic capacitance, impacting accuracy. Electromagnetic sensing is limited to specific magnetic materials, greatly restricting the range of proximity sensing. Photoelectric and ultrasonic methods face challenges with complex device integration and high-power consumption. For tactile perception of sliding, existing methods include piezoresistive [7,8], vibrational [9,10], and purely visual approaches [11,12,13,14]. These methods require external power sources, have complex structures, and are relatively costly. Therefore, there is an urgent need for a new sensing method for both proximity and sliding perception in tactile sensing.

In recent years, triboelectric nanogenerators (TENGs), based on contact electrification and electrostatic induction principles, have become a research hotspot [15,16,17,18]. TENGs are characterized by simple structures, low cost, and a wide range of material choices. Current research on TENGs focuses on micro-nano energy [19,20,21,22,23,24], self-powered systems [25,26,27,28,29,30,31,32,33,34], blue energy [35,36,37,38], and high-voltage power sources [39,40,41,42,43]. Due to the principle of electrostatic induction, TENGs can generate alternating current signals even in non-contact situations, making them suitable for proximity sensing. Additionally, by designing the electrode structure appropriately, TENGs can also sense the motion state of sliding objects, providing significant advantages for sliding perception. This offers a promising new strategy for effective proximity and sliding sensing in the field of tactile perception.

In this paper, we have developed a self-powered, tactile, integrated system that utilizes TENG for sliding and proximity sensing. Specifically, the system uses the principles of electrostatic induction and contact electrification to generate a tactile response when an external object approaches and slides. In addition, we use fluorinated ethylene propylene (FEP) as the contact electrification layer and indium tin oxide (ITO) as the electrostatic induction electrode to achieve transparency and flexibility of the entire device. By utilizing the transparency of the sensor, we can combine it with an optical camera to achieve integrated perception of touch and vision. This multimodal sensing capability provides significant advantages for applications in the field of intelligent perception. The self-powered, tactile, integrated system is expected to be integrated with different types of optical sensors to further enable multi-mode intelligent sensing and sensing technology.

## 2. Materials and Methods

### 2.1. Fabrication of the SFTTS

The size of the fluorinated ethylene propylene (FEP) layer is 6 cm × 3 cm × 50 µm, which is the total sensor substrate. Single-electrode TENG sensing electrode made of ITO is on the left square (2 cm × 2 cm), and then a PVC friction layer is used to achieve single-electrode TENG, thereby achieving proximity sensing. Then, the two rectangular ITO on the right serve as independent layers for the induction electrodes of the TENG, with dimensions of 3 cm × 1 cm, and PVC is also used as the friction layer, thereby achieving sliding sensing. The ITO is sputtered on the surface of FEP by magnetron sputtering with a thickness of 500 nm, and polyvinyl chloride (PVC) film is glued to indium tin oxide (ITO) by double-sided adhesive, where the size of PVC is 6 cm × 3 cm × 30 µm. The extraction electrode is high-temperature-resistant wire. FEP and PVC were purchased from the Polyfluorene new material Co., Ltd. (Melbourne, Australia).

### 2.2. Characterization and Measurement

Denton Discovery 635 (Moorestown, NJ, USA) is used to form an ITO electrode on one side of the FEP film. The output voltage and current of SFTTS are measured by the electrometer (Keithley 6514, Cleveland, OH, USA). The moving platform adopts a ball screw linear motor. The pressure sensor is the tension pressure weight column force sensor purchased from Ocean Precision Sensor Company (Coral Springs, FL, USA). The visual photos were taken using the iPhone camera (Apple, Shanghai, China).

## 3. Results and Discussion

**Figure 1:**
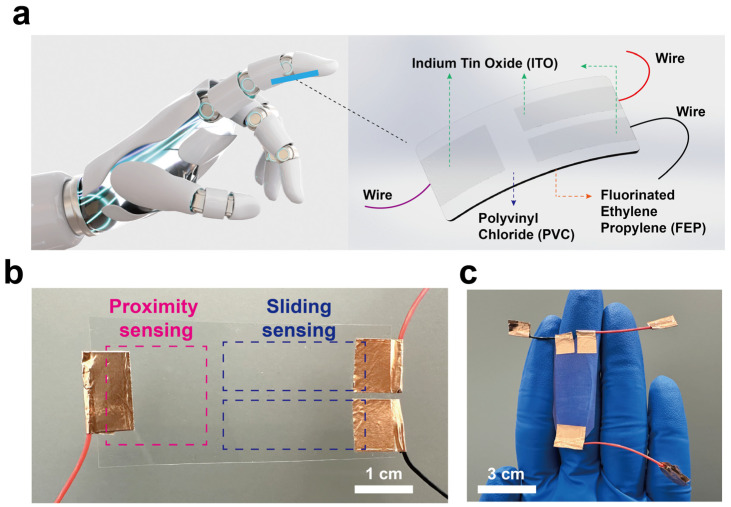
Overview of the self-powered flexible transparent tactile sensor (SFTTS). (**a**) Schematic diagram of the developed SFTTS based on triboelectric nanogenerators. (**b**) Diagram of the structural design of proximity sensing and sliding sensing parts. (**c**) A physical image of the self-powered, flexible, transparent tactile sensor attached to the finger.

Figure 1 illustrates the conceptual diagram of the SFTTS. With the advancement of intelligence and robotics, the integration of smart sensors with robotic fingers is becoming a hot topic. Due to its light, thin, and flexible characteristics, our SFTTS can be easily integrated into robotic hands to achieve proximity and tactile sensing. As shown in Figure 1a, in order to achieve device flexibility and transparency, we use FEP as the substrate material throughout the entire process. FEP has good wear resistance, a wide temperature range, and good chemical inertness, making it a commonly used substrate material for flexible devices. In addition, ITO is chosen as the electrode because it is one of the few transparent conductive materials. ITO is commonly used as an electrode in solar cells, which has good conductivity and transparency and can be uniformly coated on FEP using magnetron sputtering. PVC is chosen as the friction layer because it has strong electronegativity and is particularly suitable as the friction charge generation layer for TENG. Figure 1b shows the physical diagram of the SFTTS. It is divided into two parts: the left side is the proximity sensing area, which uses a single-electrode TENG to sense the proximity of the measured object. It is a rectangular area, and the size is 2 cm × 2 cm. The right side is the tactile sensing area, designed with an independent layer TENG to perceive the tactile sensation when the measured object slides on the friction layer surface. It is composed of two rectangular regions that are relatively close in distance; the size of one rectangular region is 3 cm × 1 cm. Since the substrate, electrode, and friction layer are all transparent, the device achieves full transparency and flexibility. Figure 1c shows the sensor attached to a finger. It can be clearly seen that the SFTTS device adheres well to the finger joints, and with a thickness of less than 100 μm, it achieves the characteristics of being light, thin, and flexible.

**Figure 2:**
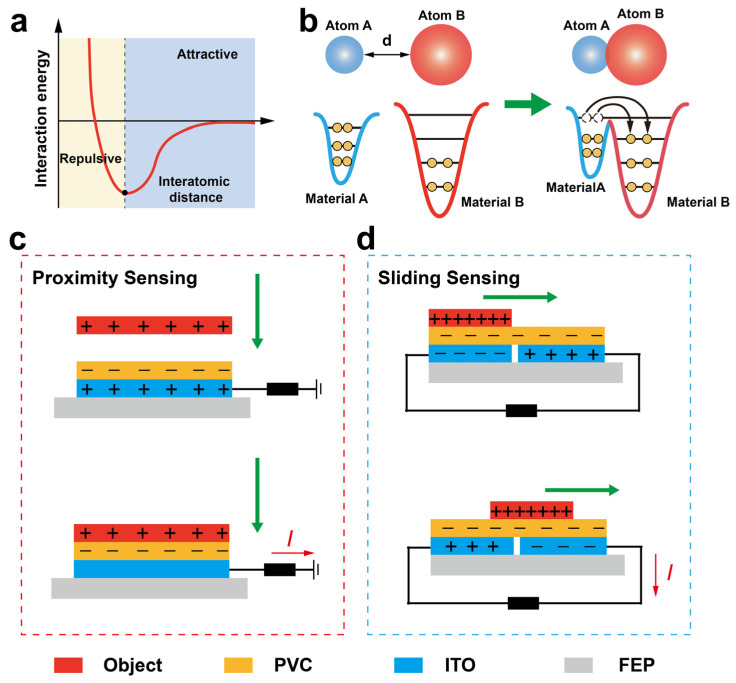
The working principle of proximity and sliding sensation using triboelectric nanogenerators. (**a**) The changes in the attractive and repulsive forces between atoms at different distances. (**b**) Electron cloud overlap and charge transfer model during materials friction. (**c**) The mechanism of single-layer triboelectric nanogenerators of proximity sensing. (**d**) The mechanism of free-standing layer triboelectric nanogenerators of sliding sensing.

Figure 2 illustrates the working principle of the SFTTS, which primarily utilizes the principles of contact electrification and electrostatic induction. Figure 2a shows the force changes at the atomic scale before and after friction between two different materials. When the distance between two atoms is relatively large, the main force between them is attractive. This attraction mainly arises from the interaction caused by the polarization of one atom by the rapidly changing electric dipole moment of another atom over time. This interaction generates an attractive force that pulls the atoms closer together. However, when the atoms are very close, the situation changes, and repulsive forces become dominant. This is because the outer electron clouds of the atoms begin to overlap, leading to repulsion between the electrons. This repulsion is due to the increased Coulomb repulsion force between the electrons caused by the overlapping electron clouds, resulting in a repulsive force that pushes the atoms apart. Therefore, the force between atoms exhibits different characteristics at different distances: attraction at long distances and repulsion at short distances. Figure 2b uses the electron cloud model to explain the charge transfer phenomenon that occurs when two atoms approach each other due to the overlapping electron clouds. Before contact, electrons are distributed in their respective orbitals in materials A and B according to the Pauli exclusion principle and energy level theory, forming two independent electron cloud potential wells. When materials A and B are in friction, the atomic distance decreases sharply, and electron cloud overlap occurs. At this time, the high-energy electrons in the potential well of material A will transfer to the low-energy level in the potential well of material B, resulting in charge transfer. This is the essence of charge generation by friction between two materials. Figure 2c illustrates the working principle of proximity sensing using a single-electrode mode TENG. When the measured object approaches the PVC friction layer from a certain distance, due to the different charges on the surface of the measured object and the material, the PVC surface usually carries a negative charge (PVC has strong electronegativity). At this time, the ITO electrode will correspondingly sense a positive charge. When the measured object is fully close to the PVC friction layer, the charges on its surface and the PVC are equal in quantity and opposite in direction, resulting in mutual cancellation. Therefore, the ITO electrode will not sense any charge. Thus, from a certain distance to full contact, there is a movement of charges between the ITO and the ground, generating a current, with the current direction from the ITO to the ground. When separated, the direction is exactly the opposite. Figure 2d shows the working principle of tactile sensing using an independent layer mode TENG. When the measured object slides on the surface of the PVC friction layer, opposite charges can be correspondingly sensed on the two independent electrodes. When the position of the measured object changes, the charges sensed by the two independent electrodes will change, generating voltage and current signals in different directions. By analyzing the direction of the electrical signal changes, the direction and position of the sliding can be determined. It is worth noting that the phenomenon of contact electrification theoretically exists between any two different materials; thus, the triboelectric nanogenerator technology has a wide range of material sensing capabilities.

**Figure 3:**
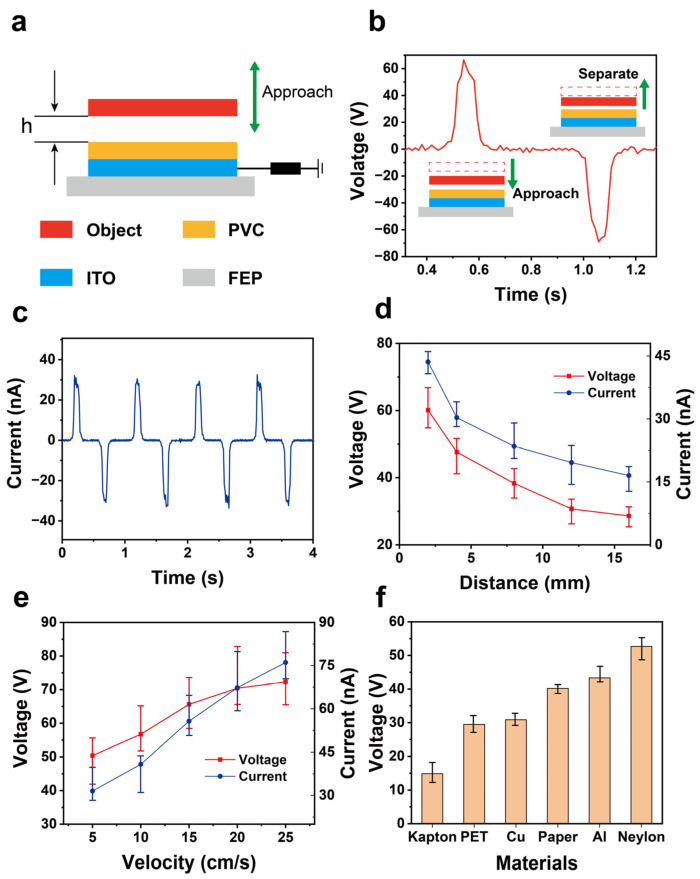
The electrical performance of the proximity sensor of SFTTS. (**a**) The motion-electrical analysis model of the proximity sensor. (**b**) The voltage output signal as the measured object approaches the sensor. (**c**) The current output signal of the measured object and the sensor at different times. (**d**) The influence of different starting distances on the voltage and current output of the proximity sensor. (**e**) The changes of voltage and current output of the proximity sensor with various velocities. (**f**) The output voltage generated by the proximity sensor for the proximity of different materials.

For the electrical performance of the proximity sensor, as shown in Figure 3a, the proximity sensor can be simplified into a model consisting of a proximity layer, a friction layer, and a sensing layer. The initial distance and movement speed affect the electrical output. Figure 3b displays the voltage waveform signal generated when the measured object approaches the sensor. It can be analyzed that a positive pulse waveform is generated during the approach, with a maximum value of approximately 70 V. When fully in contact, the voltage is 0. Subsequently, when the object moves away from the sensor, a reverse pulse voltage is generated in the circuit, confirming the possibility of determining the proximity state through the voltage waveform. As shown in Figure 3c, the movement of the measured object changes the capacitance (C) between the object and the electrode, altering the charge amount in the parallel plate capacitor, thus forming an alternating current in the external circuit with a peak value of about 30 nA. Figure 3d indicates that as the initial distance increases, the voltage and current signals generated by the proximity sensor show a decreasing trend, dropping from 60 V and 45 nA at a distance of 2 mm to 28 V and 16 nA at 16 mm. This is because the increase in distance reduces the induced charge, thereby decreasing the sensor’s output performance. Figure 3e shows that with the increase in proximity speed, the voltage and current signals significantly increase, rising from 50 V and 30 nA at 5 cm/s to 71 V and 75 nA. This is because the increase in speed leads to an increase in the amount of transferred charge, resulting in a significant increase in current. Subsequently, the voltage signals generated by different materials in contact with the PVC friction layer were measured. As is shown in Figure 3f, by measuring six materials: Kapton, PET, Cu, paper, Al, and nylon, it can be seen that Kapton has the lowest output voltage, about 15 V, while Nylon has the highest output, at 50 V. This is due to the different electronegativities of the materials. Therefore, the amplitude can be used to preliminarily determine the type of proximity material.

**Figure 4:**
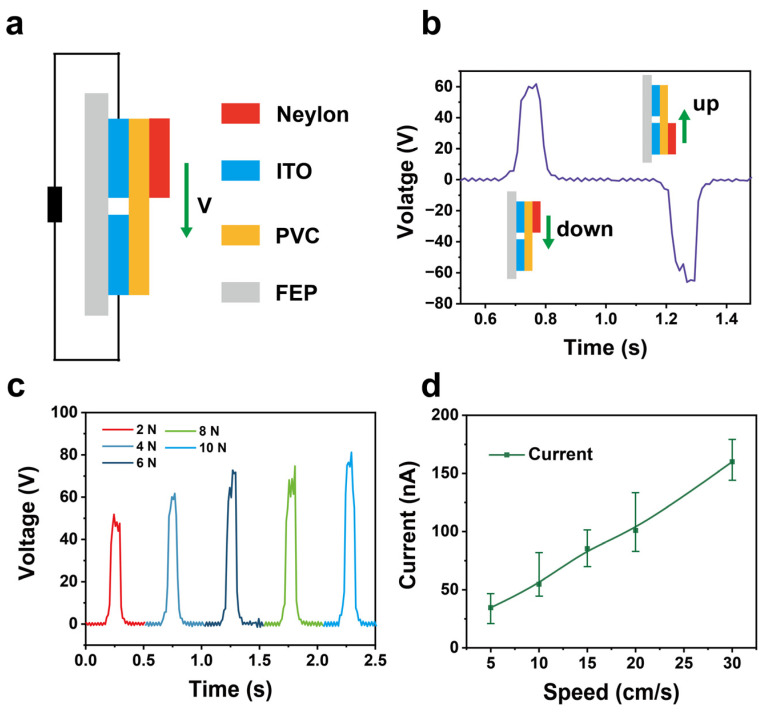
The electrical performance of the sliding sensor of SFTTS. (**a**) The working diagram of the sliding sensor. (**b**) The voltage output signal as the measured object sliding on the sensor. (**c**) The output voltage of the sliding sensor under different pressures. (**d**) The changes of current output of the sliding sensor with various velocities.

For the sensing performance of the sliding tactile sensor using an independent layer TENG, Figure 4a shows that the sliding tactile sensor can be simplified into a model consisting of a sliding layer, a friction layer, and two electrode sensing layers. When the measured object slides down the PVC surface, different charges are induced on the two ITO electrodes, generating an alternating current signal. Figure 4b indicates that when the object slides from top to bottom, a positive pulse voltage with an amplitude of 60 V is generated, while sliding from bottom to top produces a negative pulse voltage. The direction of the object’s movement can be determined by the polarity of the voltage. Figure 4c presents the output voltage under different pressures. It can be observed that as the pressure increases, the output voltage shows an upward trend, rising from 50 V at 2 N to 80 V at 10 N. This may be due to the increase in actual contact area caused by the pressure. Similarly, as is shown in Figure 4d, the current generated by the sliding tactile sensor exhibits good linear feedback with the speed increases, increasing from 40 nA at 5 cm/s to 160 nA at 30 cm/s. These results highlight the sensor’s advantages in detecting sliding position and speed.

**Figure 5:**
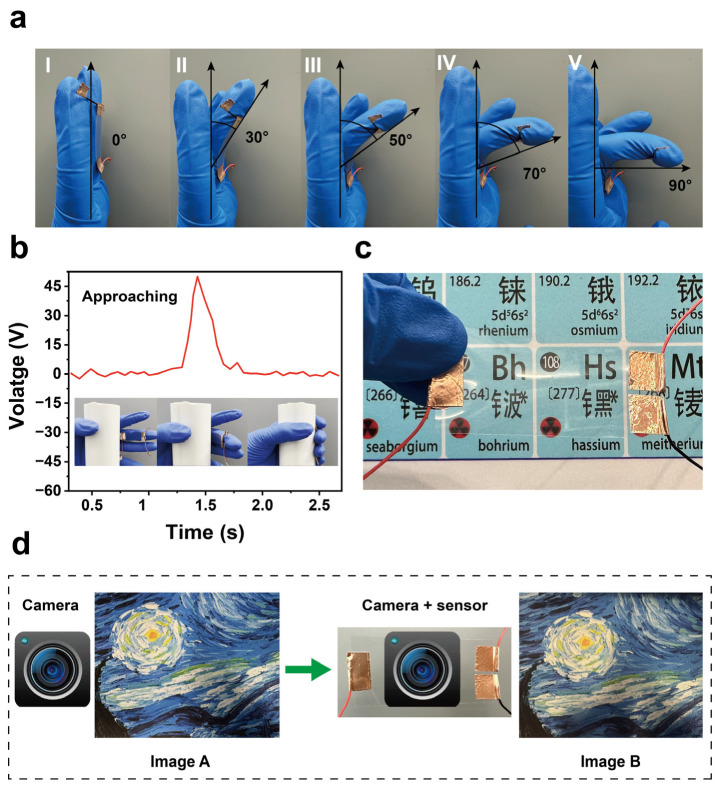
The application of the SFTTS and integration with optical sensors. (**a**) SFTTS achieves better adhesion under different bending angles (I: 0°, II: 30°, III: 50°, IV: 70°, V: 90°), which reflects the characteristics of high flexibility. (**b**) The proximity perception of gripping objects in the fingers by SFTTS. (**c**) The SFTTS made from ITO and FEP are extremely transparent in color background. (**d**) The clarity and resolution of photos taken by optical cameras without (Image A) or with (Image B) SFTTS.

Figure 5 illustrates the practical applications of the SFTTS and its integration with optical cameras. Figure 5a highlights the light, thin, and flexible characteristics of the SFTTS, which can be firmly attached to the joints of a finger. It maintains good adhesion even as the finger bends at different angles (0°, 30°, 50°, 70°, and 90°) and can achieve a maximum bending angle of 90 degrees. Figure 5b shows that when the SFTTS sensor is attached to a finger, it can achieve proximity sensing during the gripping process. The figure clearly demonstrates that when grasping a cylindrical object, the contact sensor generates a significant voltage pulse, proving the effectiveness of proximity sensing. Figure 5c demonstrates the high transparency of the SFTTS sensor. Using a colored periodic table as the background, it can be seen that the SFTTS does not affect the background color or transparency, making it well-suited for integration with optical cameras to achieve visual-tactile fusion. Figure 5d shows the results of capturing a colored image with an optical camera (as shown in Image A). Subsequently, the SFTTS sensor is integrated above the optical camera, and the colored image is captured again (as shown in Image B). Comparing Image B with Image A, it is evident that the impact on the optical camera’s imaging is minimal, with only slight color differences and no significant effect on the texture and clarity of the image. This demonstrates the significant advantage of integrating the SFTTS with visual sensors, which is of great value for multimodal sensing of robot fingers in the future.

## 4. Conclusions

In this paper, we propose a self-powered, tactile, integrated system that utilizes triboelectric nanogenerators (TENG) to achieve sliding and proximity sensing. Specifically, this system realizes tactile responses through the principles of electrostatic induction and contact electrification when external objects approach and slide.

Firstly, we use fluorinated ethylene propylene (FEP) as the contact electrification layer and indium tin oxide (ITO) as the electrostatic induction electrode, achieving transparency and flexibility for the entire device. This design makes the sensor not only powerful in function but also aesthetically pleasing.

Secondly, experimental results indicate that the material type, speed, and pressure of the measured object cause variations in the electrical signals. As the proximity distance decreases, sliding pressure increases, and both proximity and sliding speeds increase, the voltage and current of the proximity and sliding sensors based on the TENG principle significantly increase. This demonstrates that the system can not only detect the presence of objects but also identify their characteristics and states of motion.

Thirdly, by leveraging the transparency of the sensors, we can integrate them with optical cameras to achieve integrated tactile and visual perception. This multimodal sensing capability provides significant advantages for applications in the field of intelligent sensing. For example, in robotics, the combination of tactile and visual sensing can greatly enhance the robot’s environmental awareness. This self-powered, tactile, integrated system is expected to be integrated with various types of optical sensors, further realizing multimodal intelligent sensing and sensing technology.

In the future, it is believed that by combining mathematical methods such as chaos theory and fractional calculus with artificial intelligence algorithms [44], it will be possible to better analyze the shape, motion, and material characteristics of the measured objects through electrical signals. This will make important contributions to the intelligence and integration of robotic sensing, promoting the development and application of intelligent sensing technology.

## Data Availability

The original contributions presented in this study are included in the article. Further inquiries can be directed to the corresponding authors.

## References

[1] Guan F., Xie Y., Wu H., Meng Y., Shi Y., Gao M., Zhang Z., Chen S., Chen Y., Wang H. (2020). Silver nanowire–bacterial cellulose composite fiber-based sensor for highly sensitive de-tection of pressure and proximity. ACS Nano.

[2] Fonseca D., Safeea M., Neto P. (2022). A flexible piezoresistive/self-capacitive hybrid force and proximity sensor to interface collaborative robots. IEEE Trans. Ind. Inform..

[3] Cheng Y., Wang R., Zhai H., Sun J. (2017). Stretchable electronic skin based on silver nanowire composite fiber electrodes for sensing pressure, proximity, and multidirectional strain. Nanoscale.

[4] Melzer M., Mönch J.I., Makarov D., Zabila Y., Cañón Bermúdez G.S., Karnaushenko D., Baunack S., Bahr F., Yan C., Kaltenbrunner M. (2015). Wearable magnetic field sensors for flexible electronics. Adv. Mater..

[5] Ge J., Wang X., Drack M., Volkov O., Liang M., Cañón Bermúdez G.S., Illing R., Wang C., Zhou S., Fassbender J. (2019). A bimodal soft electronic skin for tactile and touchless interaction in real time. Nat. Commun..

[6] Cañón Bermúdez G.S., Karnaushenko D.D., Karnaushenko D., Lebanov A., Bischoff L., Kaltenbrunner M., Fassbender J., Schmidt O.G., Makarov D. (2018). Magnetosensitive e-skins with directional perception for augmented reality. Sci. Adv..

[7] Choi B., Choi H.R., Kang S. (2005). Development of tactile sensor for detecting contact force and slip. Proceedings of the 2005 IEEE/RSJ International Conference on Intelligent Robots and Systems.

[8] Schöpfer M., Schürmann C., Pardowitz M., Ritter H. (2010). Using a piezo-resistive tactile sensor for detection of incipient slippage. Proceedings of the ISR 2010 (41st International Symposium on Robotics) and ROBOTIK 2010 (6th German Conference on Robotics).

[9] Heyneman B., Cutkosky M.R. (2016). Slip classification for dynamic tactile array sensors. Int. J. Robot. Res..

[10] Fernandez R., Payo I., Vazquez A.S., Becedas J. (2014). Micro-vibration-based slip detection in tactile force sensors. Sensors.

[11] Ikeda A., Kurita Y., Ueda J., Matsumoto Y., Ogasawara T. (2004). Grip force control for an elastic finger using vision-based incipient slip feedback. Proceedings of the 2004 IEEE/RSJ International Conference on Intelligent Robots and Systems (IROS) (IEEE Cat No 04CH37566).

[12] Chorley C., Melhuish C., Pipe T., Rossiter J. (2009). Development of a tactile sensor based on biologically inspired edge encoding. Proceedings of the 2009 International Conference on Advanced Robotics.

[13] James J.W., Pestell N., Lepora N.F. (2018). Slip detection with a biomimetic tactile sensor. IEEE Robot. Autom. Lett..

[14] Xu Y., Zhang R., Sun J., Zhang D., Zhao Q., Duan J., Yang L. (2024). A flexible proximity-pressure–temperature tri-mode robotic sensor with stimulus discriminability, high sensitivity and wide perception range. Mater. Des..

[15] Choi D., Lee Y., Lin Z.-H., Cho S., Kim M., Ao C.K., Soh S., Sohn C., Jeong C.K., Lee J. (2023). Recent advances in triboelectric nanogenerators: From technological progress to commercial applications. ACS Nano.

[16] Wang Z.L., Wang A.C. (2019). On the origin of contact-electrification. Mater. Today.

[17] Fan F.-R., Tian Z.-Q., Wang Z.L. (2012). Flexible triboelectric generator. Nano Energy.

[18] Zou H., Zhang Y., Guo L., Wang P., He X., Dai G., Zheng H., Chen C., Wang A.C., Xu C. (2019). Quantifying the triboelectric series. Nat. Commun..

[19] Wang J., Li S., Yi F., Zi Y., Lin J., Wang X., Xu Y., Wang Z.L. (2016). Sustainably powering wearable electronics solely by biomechanical energy. Nat. Commun..

[20] Yi F., Wang X., Niu S., Li S., Yin Y., Dai K., Zhang G., Lin L., Wen Z., Guo H. (2016). A highly shape-adaptive, stretchable design based on conductive liquid for energy harvesting and self-powered biomechanical monitoring. Sci. Adv..

[21] Pu X., Liu M., Chen X., Sun J., Du C., Zhang Y., Zhai J., Hu W., Wang Z.L. (2017). Ultrastretchable, transparent triboelectric nanogenerator as electronic skin for biomechanical energy harvesting and tactile sensing. Sci. Adv..

[22] Hinchet R., Yoon H.-J., Ryu H., Kim M.-K., Choi E.-K., Kim D.-S., Kim S.-W. (2019). Transcutaneous ultrasound energy harvesting using capacitive triboelectric technology. Science.

[23] Ouyang H., Liu Z., Li N., Shi B., Zou Y., Xie F., Ma Y., Li Z., Li H., Zheng Q. (2019). Symbiotic cardiac pacemaker. Nat. Commun..

[24] Chen C., Chen L., Wu Z., Guo H., Yu W., Du Z., Wang Z.L. (2020). 3d double-faced interlock fabric triboelectric nanogenerator for bio-motion energy harvesting and as self-powered stretching and 3d tactile sensors. Mater. Today.

[25] Liu Z., Hu Y., Qu X., Liu Y., Cheng S., Zhang Z., Shan Y., Luo R., Weng S., Li H. (2024). A self-powered intracardiac pacemaker in swine model. Nat. Commun..

[26] Guo H., Pu X., Chen J., Meng Y., Yeh M.-H., Liu G., Tang Q., Chen B., Liu D., Qi S. (2018). A highly sensitive, self-powered triboelectric auditory sensor for social robotics and hearing aids. Sci. Robot..

[27] Meng K., Chen J., Li X., Wu Y., Fan W., Zhou Z., He Q., Wang X., Fan X., Zhang Y. (2019). Flexible weaving constructed self-powered pressure sensor enabling continuous diagnosis of cardiovascular disease and measurement of cuffless blood pressure. Adv. Funct. Mater..

[28] Chen J., Chen B., Han K., Tang W., Wang Z.L. (2019). A triboelectric nanogenerator as a self-powered sensor for a soft–rigid hybrid actuator. Adv. Mater. Technol..

[29] Chen J., Guo H., Wu Z., Xu G., Zi Y., Hu C., Wang Z.L. (2019). Actuation and sensor integrated self-powered cantilever system based on teng technology. Nano Energy.

[30] Liu Z., Ma Y., Ouyang H., Shi B., Li N., Jiang D., Xie F., Qu D., Zou Y., Huang Y. (2019). Transcatheter self-powered ultrasensitive endocardial pressure sensor. Adv. Funct. Mater..

[31] Zhang X., Yu M., Ma Z., Ouyang H., Zou Y., Zhang S.L., Niu H., Pan X., Xu M., Li Z. (2019). Self-powered distributed water level sensors based on liquid–solid triboelectric nanogenerators for ship draft detecting. Adv. Funct. Mater..

[32] Fu X., Xu S., Gao Y., Zhang X., Liu G., Zhou H., Lv Y., Zhang C., Wang Z.L. (2021). Breeze-wind-energy-powered autonomous wireless anemometer based on rolling con-tact-electrification. ACS Energy Lett..

[33] Xu Z., Cao L.N.Y., Li C., Luo Y., Su E., Wang W., Tang W., Yao Z., Wang Z.L. (2023). Digital mapping of surface turbulence status and aerodynamic stall on wings of a flying aircraft. Nat. Commun..

[34] Zu L., Wen J., Wang S., Zhang M., Sun W., Chen B., Wang Z.L. (2023). Multiangle, self-powered sensor array for monitoring head impacts. Sci. Adv..

[35] Li X., Xu L., Lin P., Yang X., Wang H., Qin H., Wang Z.L. (2023). Three-dimensional chiral networks of triboelectric nanogenerators inspired by metamaterial’s structure. Energy Environ. Sci..

[36] Liang X., Liu S., Lin S., Yang H., Jiang T., Wang Z.L. (2023). Liquid–solid triboelectric nanogenerator arrays based on dynamic electric-double-layer for har-vesting water wave energy. Adv. Energy. Mater..

[37] Qiu H., Wang H., Xu L., Zheng M., Wang Z.L. (2023). Brownian motor inspired monodirectional continuous spinning triboelectric nanogenerators for extracting energy from irregular gentle water waves. Energy Environ. Sci..

[38] Wang X., Ye C., Chen P., Pang H., Wei C., Duan Y., Jiang T., Wang Z.L. (2024). Achieving high power density and durability of multilayered swing-structured triboelectric nanogenerator toward marine environmental protection. Adv. Funct. Mater..

[39] Wu H., Wang S., Wang Z., Zi Y. (2021). Achieving ultrahigh instantaneous power density of 10 mw/m(2) by leveraging the oppo-site-charge-enhanced transistor-like triboelectric nanogenerator (oct-teng). Nat. Commun..

[40] Yang Z., Yang Y., Wang H., Liu F., Lu Y., Ji L., Wang Z.L., Cheng J. (2021). Charge pumping for sliding-mode triboelectric nanogenerator with voltage stabilization and boosted current. Adv. Energy Mater..

[41] Zhao Z., Zhou L., Li S., Liu D., Li Y., Gao Y., Liu Y., Dai Y., Wang J., Wang Z.L. (2021). Selection rules of triboelectric materials for direct-current triboelectric nanogenerator. Nat. Commun..

[42] Han K., Luo J., Chen J., Liu Y., Li J., Wang Z.L., Mai W. (2022). Plastic film based lightweight thruster driven by triboelectric nanogenerator for multi-purpose propulsion applications. Nano Energy.

[43] Wang Z., Jin Y., Lu C., Wang J., Song Z., Yang X., Cao Y., Zi Y., Wang Z.L., Ding W. (2022). Triboelectric-nanogenerator-enabled mechanical modulation for infrared wireless communica-tions. Energy Environ. Sci..

[44] Hernández-Acosta M.A., Martínez Gutiérrez H., Martínez-González C.L., Torres-SanMiguel C.R., Trejo-Valdez M., Torres-Torres C. (2018). Fractional and chaotic electrical signatures exhibited by random carbon nanotube networks. Phys. Scr..

